# Upregulated PARP1 confers breast cancer resistance to CDK4/6 inhibitors via YB-1 phosphorylation

**DOI:** 10.1186/s40164-023-00462-7

**Published:** 2023-11-30

**Authors:** Chuntao Quan, Zhijie Wu, Juan Xiong, Manqing Li, Yu Fu, Jiaying Su, Yue Wang, Lvwen Ning, Deju Zhang, Ni Xie

**Affiliations:** 1 Biobank, Shenzhen Second People’s Hospital, Graduate School of Guangzhou Medical University, Shenzhen, 518035 People’s Republic of China; 2 Guangdong Key Laboratory for Biomedical Measurements and Ultrasound Imaging, National-Regional Key Technology, Engineering Laboratory for Medical Ultrasound, School of Biomedical Engineering, Shenzhen University Medical School, Shenzhen, 518060 People’s Republic of China; 3 Hengyang Medical School, University of South China, Hengyang, 421001 People’s Republic of China; 4 Public Health School of Sun Yat-Sen University, Guangzhou, 510182 People’s Republic of China; 5 Laboratory Department, Shenzhen Center for Chronic Disease Control, Shenzhen, 518035 People’s Republic of China; 6 Laboratory Department, Shenzhen Baoan People’s Hospital, Second Affiliated Hospital of Shenzhen University, Shenzhen, 518035 People’s Republic of China

**Keywords:** PARP1, CDK4/6 inhibitor, Drug resistance, YB-1, Cell cycle arrest

## Abstract

**Background:**

Cyclic-dependent kinase (CDK) 4/6 kinases, as the critical drivers of the cell cycle, are involved in the tumor progression of various malignancies. Pharmacologic inhibitors of CDK4/6 have shown significant clinical prospects in treating hormone receptor-positive and human epidermal growth factor receptor-negative (HR + /HER2-) breast cancer (BC) patients. However, acquired resistance to CDK4/6 inhibitors (CDK4/6i), as a common issue, has developed rapidly. It is of great significance that the identification of novel therapeutic targets facilitates overcoming the CDK4/6i resistance. PARP1, an amplified gene for CDK4/6i-resistant patients, was found to be significantly upregulated during the construction of CDK4/6i-resistant strains. Whether PARP1 drives CDK4/6i resistance in breast cancer is worth further study.

**Method:**

PARP1 and p-YB-1 protein levels in breast cancer cells and tissues were quantified using Western blot (WB) analysis, immunohistochemical staining (IHC) and immunofluorescence (IF) assays. Bioinformatics analyses of Gene Expression Profiling Interactive Analysis (GEPIA), Genomics of Drug Sensitivity in Cancer (GDSC) and Cancer Cell Line Encyclopedia (CCLE) datasets were applied to explore the relationship between YB-1/PARP1 protein levels and CDK4/6i IC_50_. Cell Counting Kit-8 (CCK-8) and crystal violet staining assays were performed to evaluate cell proliferation rates and drug killing effects. Flow cytometry assays were conducted to assess apoptosis rates and the G1/S ratio in the cell cycle. An EdU proliferation assay was used to detect the DNA replication ratio after treatment with PARP1 and YB-1 inhibitors. A ChIP assay was performed to assess the interaction of the transcription factor YB-1 and associated DNA regions. A double fluorescein reporter gene assay was designed to assess the influence of WT/S102A/S102E YB-1 on the promoter region of PARP1. Subcutaneous implantation models were applied for in vivo tumor growth evaluations.

**Results:**

Here, we reported that PARP1 was amplified in breast cancer cells and CDK4/6i-resistant patients, and knockdown or inhibition of PARP1 reversed drug resistance in cell experiments and animal models. In addition, upregulation of transcription factor YB-1 also occurred in CDK4/6i-resistant breast cancer, and YB-1 inhibition can regulate PARP1 expression. p-YB-1 and PARP1 were upregulated when treated with CDK4/6i based on the WB and IF results, and elevated PARP1 and p-YB-1 were almost simultaneously observed during the construction of MCF7AR-resistant strains. Inhibition of YB-1 or PAPR1 can cause decreased DNA replication, G1/S cycle arrest, and increased apoptosis. We initially confirmed that YB-1 can bind to the promoter region of PARP1 through a ChIP assay. Furthermore, we found that YB-1 phosphorylated at S102 was crucial for PARP1 transcription according to the double fluorescein reporter gene assay. The combination therapy of YB-1 inhibitors and CDK4/6i exerted a synergistic antitumor effect in *vitro* and in *vivo*. The clinical data suggested that HR + /HER2- patients with low expression of p-YB-1/PARP1 may be sensitive to CDK4/6i in breast cancer.

**Conclusion:**

These findings indicated that a ‘‘YB-1/PARP1’’ loop conferred resistance to CDK4/6 inhibitors. Furthermore, interrupting the loop can enhance tumor killing in the xenograft tumor model, which provides a promising strategy against drug resistance in breast cancer.

**Supplementary Information:**

The online version contains supplementary material available at 10.1186/s40164-023-00462-7.

## Introduction

CDK4/6 kinases have been reported to be aberrantly activated in many types of tumors. CDK4/6 inhibitors (CDK4/6i), such as palbociclib, ribociclib, and abemaciclib, have been approved by the FDA and have emerged as promising treatments for patients with HR + /HER2- breast cancer. CDK4/6 kinases form a complex with D-cyclins and phosphorylated RB, and inactivated RB can release cells from the G1 checkpoint to the S-phase transition in the cell cycle [1]. Although CDK4/6i have been shown to be highly effective in treating HR + /HER2- breast cancers [2], acquired resistance frequently occurs after a period of treatment [3, 4].

It is of great significance that the identification of novel therapeutic targets facilitates the supplementation of the molecular features of CDK4/6i resistance, and these hidden molecular mechanisms can be fully beneficial in clinical practice. CDK4/6i combined with endocrine therapy is the standard of care for patients with HR + /HER2- advanced breast cancer [4–6]. Furthermore, the combination of CDK4/6i with other targeted therapies may help overcome acquired resistance, as an increasing number of potential targets for CDK4/6i resistance are being discovered. Recent studies have shown that bromodomain and extraterminal domain (BET) proteins may present new vulnerabilities after the emergence of CDK4/6i resistance, and combined BET and CDK4/6 inhibition may bring about synergistic antitumor effects in TNBC [7]. Other scholars have found that the combination of CDK4/6 and PI3K inhibition may prevent early adaptation to acquired resistance in breast cancer [8]. Recent studies have shown that combined therapy with CDK4/6i and anti-HER2 antibodies prevents tumor recurrence in a transgenic mouse model of HER2-positive breast cancer [9]. CDK4/6i combined with PARPi can inhibit tumor cell proliferation by preventing DNA repair after DNA-damaging agent treatment [10]. FGFR is also a potential target for CDK4/6i resistance, as suppression of FGFR signaling was shown to reverse CDK4/6i resistance in HR + breast cancer [11, 12]. Moreover, CDK4/6i may enhance the efficacy of immune checkpoint blockade agents, and it is possible that CDK4/6i exert anticancer effects by triggering immune activation. The CDK4/6i combined with anti-PD-1 or CTLA4 antibodies can bring about a synergistic antitumor effect [13]. Another study supported that CDK4/6i remarkably strengthened tumor immunogenicity and provided a theoretical basis for combination therapy with CDK4/6 inhibitors and immunotherapies as anticancer treatments [14]. It has been found that deletion of the tumor suppressor can also mediate CDK4/6i resistance in a series of studies. The tumor suppressor FAT1 may mediate CDK4/6i resistance in ER + breast cancer [15]. Loss of HDAC5, an obviously uncharacterized tumor suppressor protein, is an important cause of CDK4/6i resistance [16]. Another study uncovered RB and PTEN loss as a key factor in acquired CDK4/6i resistance in ER-positive advanced breast cancer [17]. However, a previous study illustrated that suppression of the tumor suppressor protein INK4 may restore CDK4/6i sensitivity [18].

We sought to investigate the molecular profiles associated with intrinsic and acquired resistance to CDK4/6i in HR + /HER2- BC. We focused on the functions of the transcription factors YB-1 and KLF5 in previous studies [19, 20]. The transcription factor YB-1 belongs to the cold-shock domain protein family and is an oncogenic transcription factor that regulates DNA transcription, DNA repair, and translation activation [21–23]. A previous study reported the key biological role of YB-1 in the occurrence and development of cancer, including tumor cell proliferation, progression, and multidrug resistance [24, 25]. In addition, phosphorylation activation at the serine 102 position was critical for the function of YB-1 [26]. Several additional phosphorylation sites on YB-1 have been identified, including Tyr281, Tyr162, Ser165, and Ser176, and each promotes the expression of distinct target gene groups [21–23]. Based on the phosphorylation state, YB-1 transfers from the cytoplasm to the nucleus, where YB-1 functions as an oncogenic transcription factor by activating the transcription of genes related to proliferation, invasion, malignant transformation, and drug resistance [27].

We planned to further explore whether YB-1 may play critical roles in CDK4/6i resistance and how YB-1 evolves during the development of CDK4/6i resistance. It has been reported that YB-1 is closely related to tumor drug resistance in a series of studies. The role of YB-1 in drug resistance has been confirmed in lung adenocarcinoma cells, and researchers have discovered that targeting the YB-1/MVP axis may help to overcome gefitinib resistance in lung adenocarcinoma patients [28]. YB-1 facilitates temozolomide (TMZ) resistance by direct activation of P53 signaling in glioma [29]. Researchers found that silencing YB-1 could overcome cell adhesion-mediated drug resistance in an AKT-dependent manner in DLBCL [30]. The results from our laboratory suggested that targeting YB-1 may sensitize ER-positive CSCs to anti-ER therapy in breast cancer [20]. There is evidence that YB-1 phosphorylation (S102) is crucial for cancer drug resistance. It has been reported that targeting YB-1 phosphorylated at S102 may overcome trastuzumab resistance by eliminating the unresponsive TIC population in BC [31]. Karen et al. reported that a high level of p-YB-1^**S102**^ confers paclitaxel resistance by inducing CD44 and CD49f expression in BC cancer cells [32]. Researchers have identified that inhibition of YB-1 phosphorylation can suppress metastasis and overcome resistance in hepatocellular carcinoma [33]. It has been reported that YB-1 phosphorylation is closely related to double strand break (DSB) repair and G1-S transition. Konstanze et al. noted that YB-1 phosphorylation (S102) enhances DSB repair in BC and that YB-1 inhibition by LJI308 may induce radiosensitization in breast cancer cells [34]. The same conclusion was confirmed that YB-1 phosphorylation plays a crucial role in DNA-DSB repair induced by ionizing radiation [35]. Further research suggested that YB-1 phosphorylation (S102) was consistent with the G1-S transition synchronized during cell cycle phases [36]. Comprehensively taking into account the function of YB-1 phosphorylation in promoting DSB repair and driving cell cycle G1/S phase transition, we hypothesize that YB-1 phosphorylation may remove the blocking effect of CDK4/6i on G1/S phase transition and lead to the occurrence of CDK4/6i resistance in breast cancer.

## Materials and methods

### Cell culture and inhibitors

SK-BR-3, MCF7, MDA-MB-231, HCC1937, T47D and HCC1806 cells were obtained from ATCC. MCF7AR was generated from the parent MCF7 cell by treating abemaciclib continuously. All cells were cultured in DMEM (Gibco) supplemented with 10% fetal bovine serum and 1% antibiotic (Gibco) at 37 °C with 5% CO2. Cells were used for no longer than 12 months before being replaced. Abemaciclib and olaparib were purchased from TargetMol, USA, and LJI308 was purchased from Med Chem Express, USA. The inhibitors were dissolved in DMSO.

### Generation of abemaciclib-resistant cell lines

MCF7 abemaciclib-resistant cells (MCF7AR) were derived by treating MCF7 cells with increasing concentrations of abemaciclib starting at 0.1 μM, followed by a stepwise dose up to a final abemaciclib concentration of 20 μM. On the 1st day, the MCF7 cells were exposed to 0.1 μM abemaciclib for 3 days, and then the cells were grown in medium without drugs. The density of the remaining cells returned to 80% after approximately 4 days. On the 8th day, the cells were cultured in medium with 0.3 μM abemaciclib for 3 days and recovered to 80% confluence in the next 7 days. On the 18th day, we increased the drug concentration to 1 μM for 5 days of treatment and recovered to 80% density in the next 7 days. On the 30th day, we exposed the cells to 1 μM abemaciclib in media for up to 30 days, and the cells grew steadily in media with 1 μM abemaciclib. On the 60th day, we increased the abemaciclib concentration to 5 μM for approximately 3 days, and the cells recovered to 80% confluence after withdrawing the drug in the next 7 days. We exposed the cells to 5 μM abemaciclib in media for the next 20 days. On the 90th day, the cells continued to grow in 10 μM abemaciclib for approximately 30 days. On the 120th day, the cells were exposed to 20 μM abemaciclib in media for up to 60 days. MCF7AR cells can grow stably in medium with abemaciclib at 20 μM. Cells were washed with PBS 3 times after withdrawing the drugs and replacing them with fresh drug and media. The resistant cell lines established in this manner were then maintained in drug-free medium or with 1 μM abemaciclib within 3 generations after thawing from storage in liquid nitrogen.

### Human tissue specimens

Paraffin-embedded breast cancer tumor specimens from 90 patients were collected at the First Affiliated Hospital of Shenzhen University (Shenzhen, China). The study was approved by the Institutional Review Board of First Affiliated Hospital of Shenzhen University; human sample collection procedures were in accordance with the established guidelines. All human breast cancer sample acquisitions were approved by the Committee on Ethics of Shenzhen Second People’s Hospital, Shenzhen University. The pathological information of clinical breast cancer patients is listed in Additional file 1: Table S1.

### Cell counting kit-8 (CCK-8) assays and crystal violet staining assay

CCK-8 assays were used to test the drug efficiency on the cell lines. The breast cancer cells were laid on a 96-well dish starting at 5 × 10^3^ cells per well for the CCK-8 assay. Thereafter, 10 μL CCK-8 working solution (Beyotime) was added at the indicated time points after the cells were treated with the inhibitors. The plates were incubated at 37 °C for 2 h. Next, the cell viability was measured by a microplate reader (Thermo Fisher) with the optical density of each well at 450 nm. A cell crystal violet staining assay was employed to detect cell viability based on the amounts of cell stains. Cells were laid on a 12-well dish starting at 5 × 10^4^ cells/well, and 3 parallel dishes were used to assess the experimental accuracy. The cells were all stained with crystal violet solution and then rinsed for 5 min. Next, pictures were captured under a microscope.

### *Western blot (WB) and immunohistochemistry *(*IHC)*

WB and IHC assays were performed as previously reported by our laboratory [20]. Briefly, total intracellular protein was extracted using RIPA lysis buffer (Beyotime, China) for WB assays, protease inhibitor cocktail (Beyotime) and 0.1 mM PMSF (Beyotime) were added, and a BCA assay kit (Beyotime) was used to assess protein concentrations. Thereafter, protein loading, electrophoresis and membrane transfer were performed according to the manufacturer’s instructions. Next, milk blocking, membrane washing and primary antibody incubation overnight were performed in sequence. Finally, after incubation with the secondary antibody for 2 h, the target proteins on the membrane were detected by the Beyo ECL Star Kit (Beyotime), and Bio-Rad Quantity One Software was used for quantification. The IHC assay was performed to detect PARP1 and p-YB-1 expression in human breast cancer sections and adjacent tissues. The slides were soaked in xylol for 10 min and washed in a series of alcohols with decreasing concentrations. Antigen retrieval was performed when the sections were soaked in citrate buffer in a microwave for 5 min after tissue deparaffinization. Thereafter, the sections were blocked in 1% peroxidase and then incubated in goat serum. Primary antibodies against PARP1 and YB-1 were incubated for 12 h. The biotinylated rabbit anti-mouse antibody (Vector, Germany) was used for signal amplification. Subsequently, the slides were stained with diaminobenzidine (DAB) (Sigma, USA) to label PARP1/p-YB-1 and counterstained with hematoxylin to label nuclei. To increase the accuracy of the results, the staining results came from the same position of successive slices. The antibody information for Western blot and immunohistochemistry are listed in Additional file 2: Table S2.

### Plasmid and shRNA/siRNA primers

Construction of the PARP1 and WT/S102A/S102E YB-1 plasmids were designed and synthesized by the Yunzhou Biotechnology Company in China. The wild-type and mutant YB-1 plasmids were transfected into MCF7 cells using Lipofectamine 3000 reagent (Gibco) according to the manufacturer’s instructions. The siRNAs targeting PARP1 and YB-1 were purchased from Tsingke Company (Beijing, China), and the construction of shYB-1 stable cell lines was completed by laboratory colleagues previously [20]_**.**_ The siRNAs targeting PARP1 and YB-1 were transfected into MCF7 and MCF7AR cells using Lipofectamine 3000 reagent (Gibco). The primer information is listed in Additional file 3: Table S3.

### ChIP assay and quantification of mRNA by real-time PCR

The ChIP samples from MCF7AR-siYB-1 cells were fixed with 37% formaldehyde (final concentration 1%) and then decrosslinked by adding 10 × 1.25 M glycine. DNA‒protein complexes were obtained under ultrasonication and were used for subsequent DNA purification steps. The ChIP assay (Beyotime P2080S) protocol can guide the follow-up experimental procedure according to the manufacturer’s instructions. Thereafter, a primary antibody targeting YB-1 was coincubated with the lysate of the DNA‒protein complex for immunoprecipitation, and an IgG antibody was used as a negative control. The immunoprecipitated DNA was treated with RNase A and proteinase K, followed by purification using phenol‒chloroform extraction and ethanol precipitation. The content determination of targeted DNA was analyzed by quantitative real-time PCR. The primer sequences and antibodies used in this study are listed in Additional files 2, 3: Tables S2, S3.

### Double fluorescein reporter gene assay

Construction of the reporter plasmid pGL3-Basic containing the promoter region 2100 bp of PARP1 was conducted by Tsingke in China. Renilla luciferase reference plasmid pRL-TK, pGL3-Basic and WT/S102A/S102E-YB-1 plasmid were simultaneously transfected into cells for 48 h, and empty plasmid pGL3-Basic was used as a control. PLB buffer was added for cell lysis, and then 10 μL of supernatant was transferred to 96-well wells. Next, 100 μL luciferase assay reagent was added, and the intensity of the fluorescein reaction was detected in a dark environment. Thereafter, the luciferase reaction intensity of Renilla pRL-TK was detected after adding 100 μL Stop and Glo Reagent. Three parallel wells were used to reduce errors, RLU1 represented firefly fluorescein plum intensity, and RLU2 represented Renilla luciferase intensity. The ratio of RLU1/RLU2 was further analyzed.

### Immunofluorescence and multiple immunofluorescence assays

Relevant experimental steps were carried out according to the procedure described in this paper [37]_**.**_ For the immunofluorescence assay, on Day 1, the breast cancer cells were grown on glass coverslips in 6-well plates overnight. On Day 2, we washed the slides with PBS for approximately 5 min three times, and the climbing tablet was fixed with 4% paraformaldehyde for 15 min. Then, the slides were permeabilized for 20 min with 0.5% Triton X-100, and goat serum was used for blocking at room temperature for 30 min. Finally, the first antibody was incubated overnight. On Day 3, fluorescent secondary antibody was added after 3 rinses with PBST(PBS-Tween20) in a darker environment, and then DAPI was added to restain the cell nucleus for 5 min. Finally, after the sealing solution containing the fluorescence quencher was sealed on slides, pictures were captured under a fluorescence microscope. For multiple immunofluorescence staining, an iterative staining method was used. Three rounds of staining were as follows: round 1, primary Ab-secondary Ab HRP-conjugated -TSA-fluorophore (FITC, Cy3, Cy5.5) was performed according to the three-step staining protocol. Round 2 was followed by an Ab denaturation (stripping) step and, in turn, by the next round of staining with another primary antibody. The primary and HRP-conjugated secondary Abs of the previous round were removed in the denaturation step, the remaining TSA fluorophore was guaranteed for sequential staining steps, and nuclei were visualized with DAPI. The antibody information for the immunofluorescence assay is listed in Additional file 2 Table S2.

### EdU-cell proliferation detection

EdU, a thymidine analog, can incorporate replicated DNA molecules by replacing T-thymine during cell proliferation. A stable triazole ring is formed by a fluorescently labeled covalent reaction. The effects of inhibitors such as olaparib and LJI308 on DNA replication can be analyzed quantitatively. Then, 100 μL of 50 μM EdU solution was added to the cells in the 96-well plate for 2 h of incubation and washed with PBS solution 3 times for 5 min each time. Thereafter, 50 μL glycine was coincubated with cells in each well for 5 min and then washed with PBS 3 times. Next, 100 μL of 0.5% Triton X-100 was added to enhance cell membrane permeability. Finally, 100 μL Hoechst 33,342 solution was coincubated with cells for DNA staining. All stained cells and pictures were captured by fluorescence microscopy.

### Flow cytometry and cell cycle assay

The distribution of cells at every G1/S/G2 stage was analyzed by flow cytometry. The sample DNA was labeled by the nucleic acid dye PI, which is a fluorescent nucleic acid dye. PI can bind selectively between the bases of nucleic acid DNA and RNA double helices. The binding amount is positively correlated with DNA content. MCF7AR cells were collected for the detection of the apoptosis ratio by staining with Annexin V-FITC and PI, and the operation was performed according to the instructions of the Apoptosis Detection Kit I (Beyotime, Shanghai, China). For cell cycle analysis, MCF7AR cells were fixed in 70% ethanol overnight and then stained with 20 μg/mL propidium iodide (PI) (Thermo Fisher Scientific) for 30 min. Fluorescence detection was performed by flow cytometry, and the cells were suspended before loading. The data were recorded after the signal was stabilized, and the maximum PI excitation wavelength was 535 nm. All stained cells were analyzed on a BD Flow Cytometer (BD Biosciences, San Jose, CA, USA). Data were analyzed by FlowJo software (Treestar Inc.).

### Nude mouse xenograft

The nude mice were purchased from Beijing Huafukang Company. MDA-MB-231 or MCF7AR cell mixtures with 100 μL serum-free DMEM (Gibco) were subcutaneously injected into 4 week-old BALB/c nude mice (six mice/group). The cell number for inoculation was calculated to be approximately 2 × 10^6^. The tumor volumes (mm^3^) were calculated according to the following formula: V = 1/2 ab^2^, where a represents the largest tumor diameters and b represents the shortest. Abemaciclib and olaparib were dissolved in carboxymethylcellulose sodium (CMC-Na) solution, and LJI308 was dissolved in 5% DMSO + 30% PEG300 + 10% Tween 80 solution. Abemaciclib and olaparib were administered intragastrically with 100 mg/kg injection per nude mouse, and LJI308 was administered intraperitoneally with 10 mg/kg injection. These drugs were administered three times a week. All in vivo experiments were approved by the Institutional Animal Care and Use Committee of Shenzhen University (SZU-IACUC-2022–00136) and followed the Guide for the Care and Use of Laboratory Animals.

### Statistical analysis

The results are expressed as the mean ± SEM and were analyzed using a two-tailed Student’s t test, two-way analyses of variance and ANOVA. ANOVAs were performed as previously reported [38–40]. The synergistic effects of both drugs involved in Fig. 2D–G were calculated by CompuSyn software, and the smaller the composite index (CI) values were, the better the combination effect. Pearson correlation coefficient analysis was performed using GraphPad Prism V5.0. Differences were considered statistically significant when P < 0.05.

## Results

### CDK4/6i treatment led to PARP1 upregulation in breast cancer patients and cells.

**Fig. 1:**
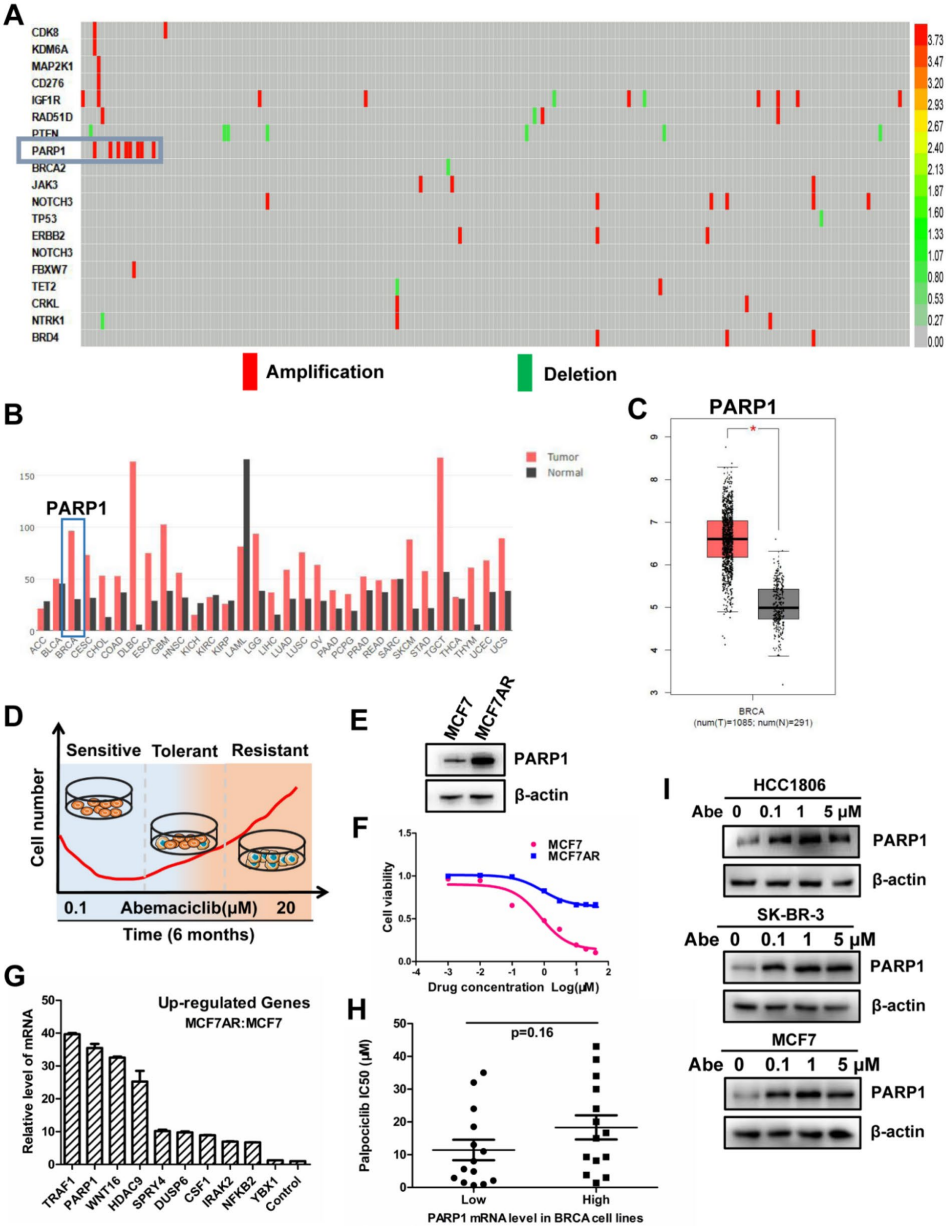
CDK4/6i treatment led to PARP1 upregulation in breast cancer patients and cells.** A**. PARP1 is upregulated in HR + breast cancer patients, who ultimately experience disease progression. **B–C**. PARP1 is highly expressed in most cancers, especially in breast cancer, according to the GEPIA database. **D**. The construction process of drug-resistant strains is shown in the picture. **E–F**. MCF7AR cell lines were confirmed to be resistant to abemaciclib with an IC_50_ of approximately 50 μM, and PARP1 was significantly upregulated in MCF7AR cells compared with parent MCF7 cells. **G**. The results of transcriptome sequencing showed that PARP1 was upregulated significantly, with a 40-fold change in MCF7AR compared with the MCF7 group. **H**. The IC_50_ for the CDK4/6i palbociclib in 30 breast cancer cell lines was obtained from the GDSC database and paired with corresponding PARP1 mRNA level data obtained from the CCLE database. The 30 breast cancer cell lines were divided into two groups based on the level of PARP1 mRNA, and those with a high level of PARP1 demonstrated a tendency to have a higher palbociclib IC_50_. **I**. Western blot results showed that Abemaciclib treatment led to PARP1 elevation in 3 breast cancer cell lines. The results presented have been repeated in 3 biological replicates. Data, means ± SEMs, *, P < 0.05, **, P < 0.01, ***, P < 0.001

To further explore what molecules were involved in the development of CDK4/6i resistance, we analyzed genomic alterations in 348 ER + breast cancers treated with CDK4/6i from the open literature published in 2018 *CancerCells* [15] and found that the protein and RNA of PARP1 were upregulated in some patients (Fig. 1A). In addition, PARP1 was highly expressed in breast cancer samples included in the GEPIA database (Fig. 1B–C). To better explore the underlying CDK4/6i resistance mechanism, we generated CDK4/6i Abemaciclib resistant cell lines (AR, Abemaciclib resistant), and the construction process of drug-resistant strains is shown (Fig. 1D). Western blot results indicated that PARP1 was significantly upregulated in AR cell lines compared with parent MCF7 cells (Fig. 1E). MCF7AR cells were confirmed to be resistant to abemaciclib with an IC_50_ of 50 μM compared with MCF7 cells with an IC_50_ of 1 μM (Fig. 1F). The results of transcriptome sequencing showed that PARP1 was upregulated significantly, with a 40-fold change in MCF7AR cells compared with MCF7 cells, and there was slight upregulation of the YBX-1 gene, which is considered to be the key regulator of PARP1 (Fig. 1G). Thus, high PARP1 protein levels may be related to CDK4/6i resistance in breast cancer. To confirm the relationship between PARP1 and CDK4/6i resistance over a wider range, we obtained the IC_50_ for the CDK4/6i palbociclib in 30 breast cancer cell lines from the GDSC database and paired them with corresponding PARP1 mRNA level data gained from CCLE. The 30 breast cancer cell lines were divided into two groups based on the level of PARP1 mRNA, and those with a high level of PARP1 demonstrated a tendency to have a higher palbociclib IC_50_ (Fig. 1H). Furthermore, Abemaciclib treatment led to PARP1 elevation in 3 breast cancer cell lines (Fig. 1I).

### PARP1 inhibition restored CDK4/6i sensitivity.

**Fig. 2:**
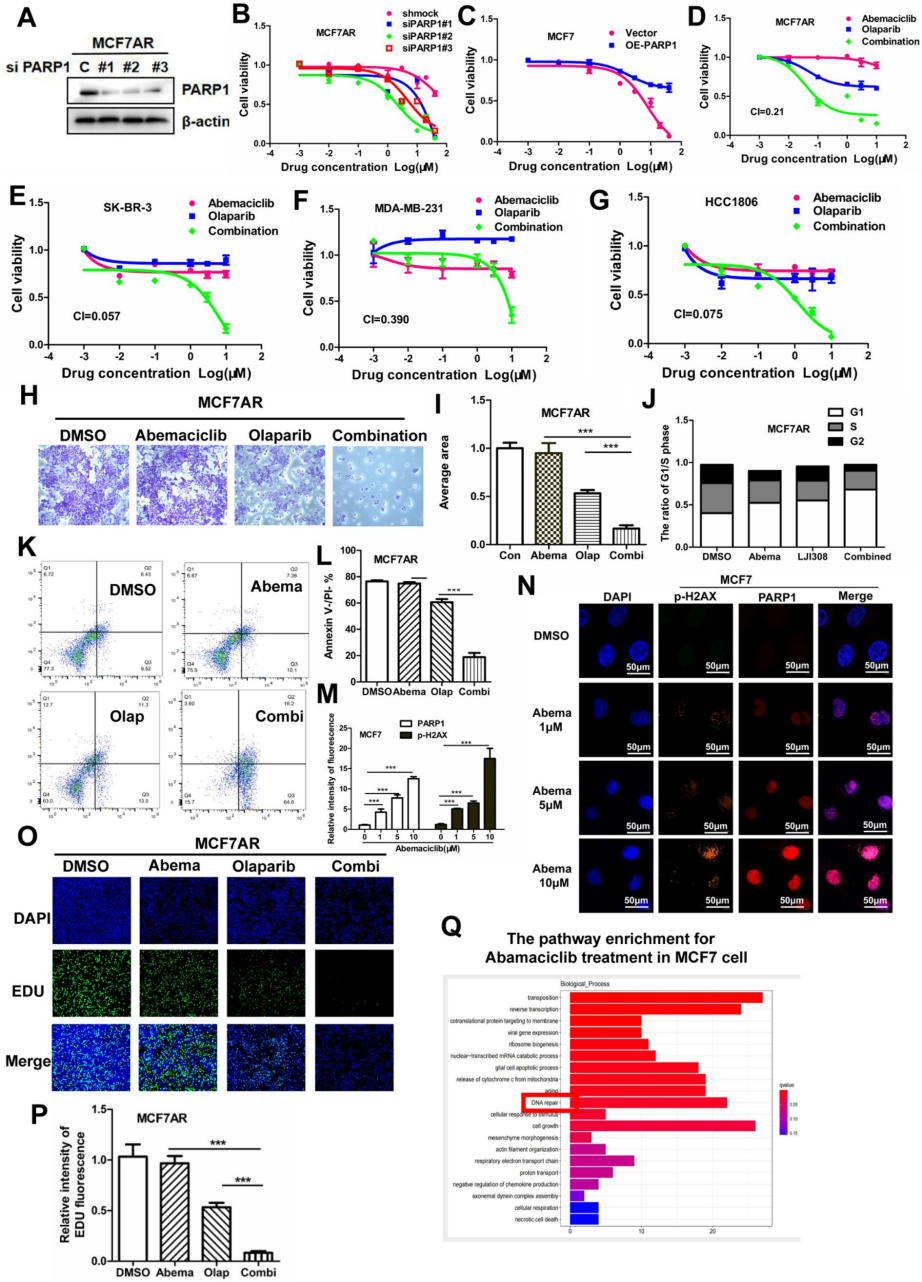
PARP1 inhibition restored CDK4/6i sensitivity. **A**–**B**. The 3 siRNA sequences in MCF7AR cells showed that PARP1 silencing restored sensitivity to abemaciclib. C. Overexpression of PARP1 restored abemaciclib sensitivity. **D–G**. Olaparib treatment promoted cell sensitivity to abemaciclib in 4 breast cancer cell lines. **H–I**. The combined therapy showed a synergistic antitumor effect by crystal violet staining assay. **J**. The cell cycle assay showed that G1 phase increased remarkably when treatment with combined therapy. **K–L**. The combined therapy induced cell apoptosis significantly in the flow cytometry assay. **M–N.** The fluorescence level of p-H2AX was upregulated simultaneously with PARP1 levels in MCF7 cells treated with abemaciclib. Orange represents p-H2AX, red represents p-YB-1 and blue represents DAPI. **O–P**. EdU can incorporate the replicated DNA molecule by replacing T-thymine during cell proliferation. **Q**. The enrichment in the DNA repair pathway when MCF7 cells were treated with abemaciclib. The results presented have been repeated in 3 biological replicates. Data, means ± SEMs, *, P < 0.05, **, P < 0.01, ***, P < 0.001

PARP1 inhibition may help us determine whether PARP1 plays a crucial role in abemaciclib resistance. We used three siRNA sequences to silence the *PARP1* gene in MCF7AR cells. Western blot analysis showed that PARP1 knockdown was successful, and the MTT assay indicated that PARP1 silencing restored abemaciclib sensitivity (Fig. 2A–B). Overexpression of PARP1 in MCF7 cells can enhance abemaciclib resistance (Fig. 2C). Olaparib, the FDA-approved drug targeting PARP1, can restore CDK4/6i sensitivity. The MTT assay indicated that olaparib can significantly decrease cell viability when combined with abemaciclib in 4 breast cancer cell lines, and the smaller the composite index (CI) values were, the better the synergistic effect. (Fig. 2D–G). The same result was obtained according to the crystal violet staining assay (Fig. 2H–I). The cell cycle assay showed that G1 phase increased remarkably when treated with combined therapy, which indicated that the cell cycle was blocked in the G1 phase (Fig. 2J). The flow cytometry results showed that the combined therapy significantly induced cell apoptosis (Fig. 2K–L). The combined therapy of abemaciclib and olaparib synergistically suppressed tumor growth in the xenograft model, and samples derived from animal tumors were used to confirm the key role of PARP1 in CDK4/6i resistance by immunohistochemical assay (Fig. 6A–C, G). The fluorescence level of p-H2AX, a DNA-damage marker molecule, was upregulated simultaneously with DNA repair protein PARP1 levels in MCF7 cells when treated with abemaciclib. The results showed that DNA damage and repair occurred synchronously when treated with abemaciclib, and the upregulation and nuclear localization of YB-1 may be correlated with abemaciclib resistance (Fig. 2M–N). The results showed that olaparib treatment induced DNA replication reduction, which may be attributed to cell cycle arrest. The combined treatment of abemaciclib and olaparib led to DNA replication stagnation (Fig. 2O–P). The DNA repair pathway was enriched when MCF7 cells were treated with abemaciclib (Fig. 2Q).

### YB-1 inhibition decreased the PARP1 level and mediated CDK4/6i sensitivity

**Fig. 3:**
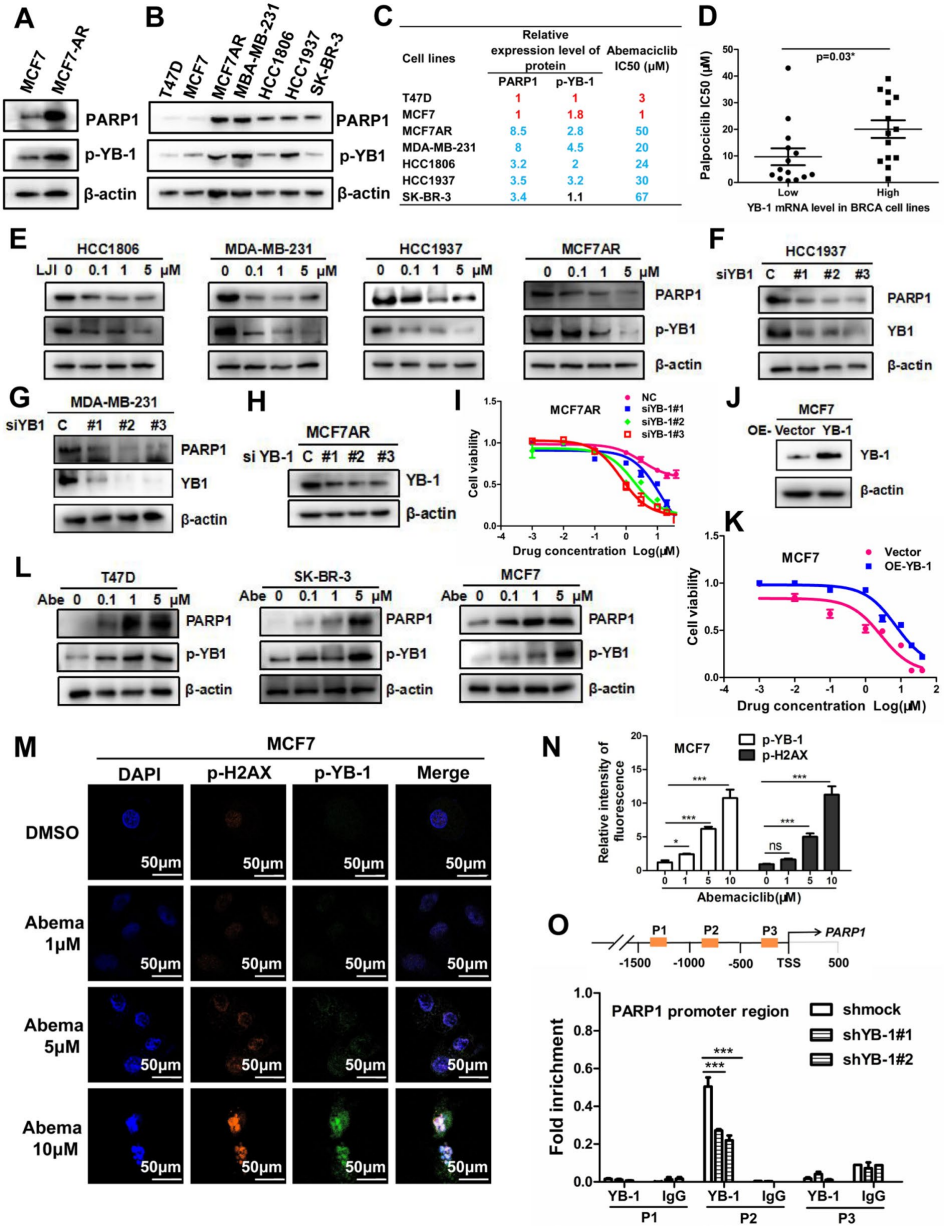
YB-1 inhibition decreased the PARP1 level and mediated CDK4/6i sensitivity. **A**. p-YB-1 was elevated in MCF7-AR cells compared with parental MCF7 cells, and the protein levels of p-YB-1 and PARP1 were positively correlated. **B-C**. The expression of p-YB-1 and PARP1 showed the same trend in seven breast cancer cell lines, and p-YB-1/PARP1 was positively correlated with the Abemaciclib IC_50_. **D**. Comparative analysis suggested that the YB-1 mRNA level was positively correlated with the CDK4/6i IC_50_. **E**. PARP1 was markedly reduced when cells were treated with the YB-1 inhibitor LJI308, which can decrease the level of p-YB-1. **F–H**. The PARP1 protein level decreased when YB-1 was silenced. **I**. YB-1 silencing increased CDK4/6i sensitivity, and the inhibitory effect was confirmed by YB-1 silencing. **J–K**. YB-1 overexpression enhanced CDK4/6i resistance in MCF7AR cells according to the MTT assay. **L**. p-YB-1 and PARP1 were positively correlated and upregulated consistently in the three cell lines treated with abemaciclib. **M–N**. The fluorescence levels of p-H2AX and p-YB-1 were increased simultaneously in MCF7 cells treated with abemaciclib and reached their maximum when treated with abemaciclib at 10 μM. Orange represents p-H2AX, green represents p-YB-1 and blue represents DAPI. **O**. Three sequence primers for the promoter region of PARP1 were used for the ChIP assay, and the data demonstrated that YB-1 binds to the P2 primer in the promoter region of PARP1. The IgG antibody was used as a negative control. The results presented have been repeated in 3 biological replicates. Data, means ± SEMs, *, P < 0.05, **, P < 0.01, ***, P < 0.001

We investigated the reason that the PARP1 protein level was elevated in the MCF7AR cell lines or abemaciclib treatment. Interestingly, we discovered that p-YB-1 was elevated in MCF7-AR cells compared with parental MCF7 cells, and p-YB-1 and PARP1 were positively correlated based on western blot results (Fig. 3A). The expression of p-YB-1/PARP1 and the abemaciclib IC_50_ were positively correlated in seven breast cancer cell lines based on the quantitative analysis results of the protein level of p-YB-1/PARP1 and IC_50_ detection (Fig. 3B–C). Comparative analysis suggested that the YB-1 mRNA level was positively correlated with the CDK4/6i IC_50_ (Fig. 3D). Previous reports have suggested that LJI308 is a YB-1 inhibitor based on the conclusion that RSK2 can directly regulate YB-1 phosphorylation [41]. PARP1 was markedly reduced when p-YB-1 was inhibited by LJI308 (Fig. 3E). In addition, the PARP1 protein level decreased significantly when YB-1 was knocked down (Fig. 3F–H). YB-1 silencing can enhance CDK4/6i sensitivity, and the inhibitory effect was confirmed by siYB-1 (Fig. 3H–I). The MTT assay confirmed that YB-1 overexpression increased CDK4/6i resistance in MCF7AR cells. Western blot data showed that YB-1 overexpression was successful (Fig. 3J–K) and that PARP1 may be regulated by YB-1 phosphorylation. In contrast, p-YB-1 and PARP1 increased consistently when breast cancer cell lines were treated with abemaciclib (Fig. 3L). The fluorescence levels of p-H2AX and p-YB-1 were upregulated simultaneously in MCF7 cells treated with abemaciclib, and the fluorescence intensity of p-H2AX and p-YB-1 reached its maximum when abemaciclib treatment was 10 μM. p-YB-1 may be correlated with DNA damage repair, which may cause CDK4/6i resistance (Fig. 3M–N). Considering that YB-1 is a transcription factor, we designed three sequence primers for the promoter region of PARP1, and a ChIP assay was employed to evaluate whether YB-1 can bind to the promoter region of PARP1. The data demonstrated that YB-1 binds to the P2 primer in the promoter region of PARP1, which may be transcriptionally activated by YB-1 (Fig. 3O).

### YB-1 inhibition weakened CDK4/6i resistance

**Fig. 4:**
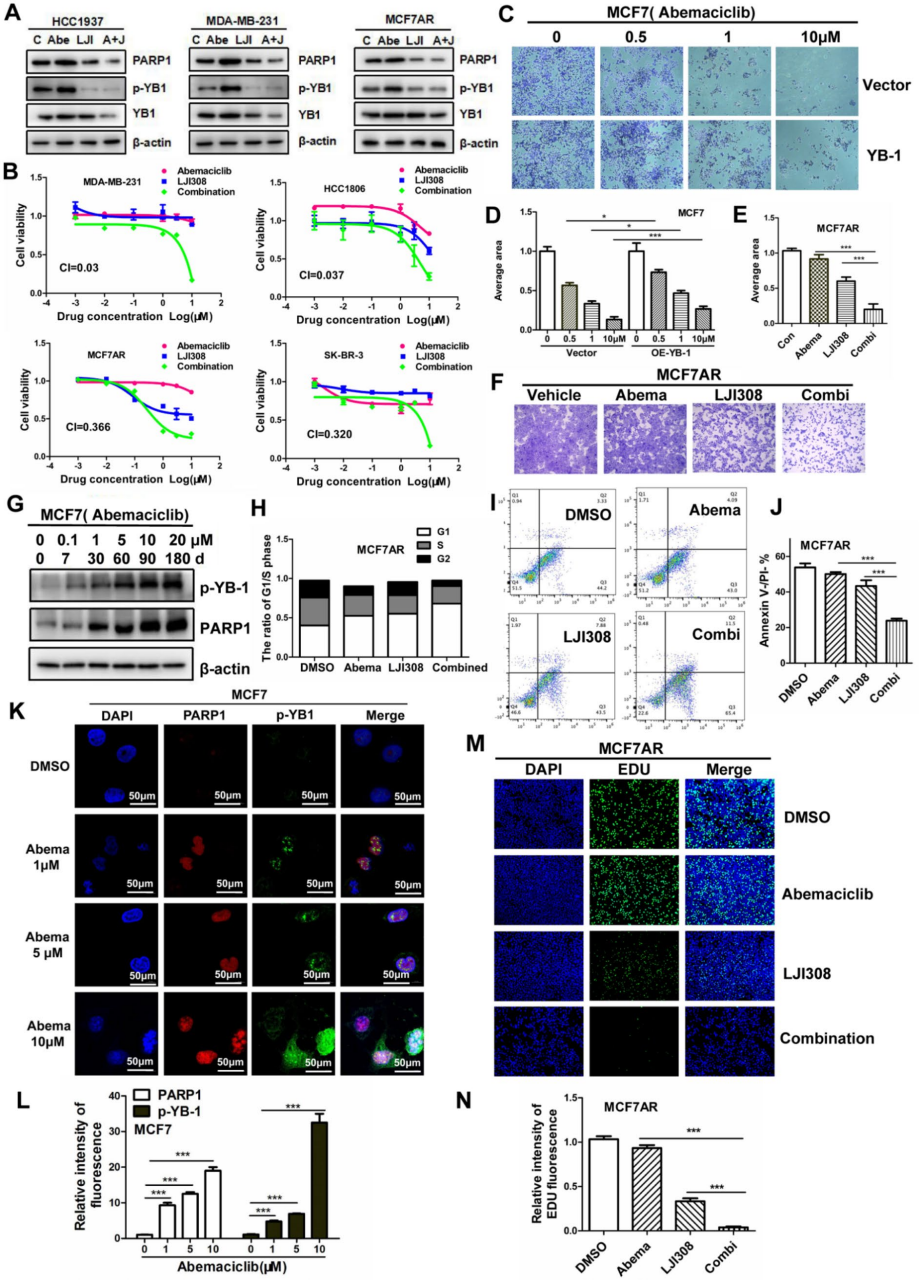
YB-1 inhibition weakened CDK4/6i resistance. **A**. PARP1 and p-YB-1 were decreased when treated with abemaciclib and LJI308 in three breast cancer cell lines. **B**. MTT assay was used to assess the cytotoxic killing effect of combined therapy with abemaciclib and LJI308 in four cell lines. **C–D**. The YB-1 plasmid was transfected into MCF7 cells, and the inhibitory effect of abemaciclib in the YB-1 group was slower than that in the control group. **E–F**. MCF7AR cells were treated with abemaciclib and LJI308, and the combined inhibitory effect was confirmed by the crystal violet staining assay. **G**. We selected abemaciclib-treated MCF7 cells from six time points during the AR screening process. Elevated PARP1 and p-YB-1 levels were observed almost as simultaneously as MCF7 cells gradually gained resistance to abemaciclib. **H**. The cell cycle assay showed that G1 phase increased remarkably when treated with combined therapy of abemaciclib and olaparib. **I–J**. The cell apoptosis induced by the combined therapy of abemaciclib and LJI308 was assessed by flow cytometry, and the result analysis statistics are shown. **K–L**. The PARP1 and p-YB-1 proteins were detected by antibody when abemaciclib treatment was performed by immunofluorescence assay. The merged image shows their colocalization in the nucleus. Red represents PARP1, green represents p-YB-1 and blue represents DAPI. **M–N**. The lower fluorescence intensity represents attenuated DNA replication, and DNA replication stagnation was induced by the combined treatment of abemaciclib and LJI308 for the EDU assay. The results presented have been repeated in 3 biological replicates. Data, means ± SEMs, *, P < 0.05, **, P < 0.01, ***, P < 0.001

We focused on whether YB-1 inhibition can enhance sensitivity to CDK4/6i. Western blot data showed that PARP1 and p-YB-1 were decreased simultaneously when treated with abemaciclib and LJI308 (Fig. 4A). As shown by the MTT assay, combined therapy with abemaciclib and LJI308 led to a cytotoxic killing effect in the four cell lines (Fig. 4B). The inhibitory effect of abemaciclib in MCF7 cells was decreased by overexpression of YB-1 compared with the control group (Fig. 4C–D). The sensitivity of Abemaciclib was augmented significantly when MCF7AR cells were treated with LJI308, based on crystal violet staining (Fig. 4E–F). These results suggested that PARP1 and p-YB-1 change simultaneously. To explore the changes in PARP1 and p-YB-1 levels during the construction of resistant strains, we used a stepwise dose-increasing (from 0.1 to 20 μM) drug treatment for 6 months. The initial drug concentration of abemaciclib was 0.1 μM. We selected abemaciclib-treated MCF7 cells from six time points during the AR screening process, and elevated PARP1 and p-YB-1 were observed almost simultaneously as MCF7 cells gradually gained resistance to abemaciclib (Fig. 4G). The cell cycle assay showed that G1 phase increased remarkably when treated with the combined therapy of abemaciclib and olaparib (Fig. 4H). Flow cytometry showed that combination therapy with abemaciclib and LJI308 was effective (Fig. 4I–J). We discovered that the PARP1 and p-YB-1 proteins were colocalized in the nucleus under abemaciclib treatment by immunofluorescence assay, and the protein level of PARP1 gradually increased in a dose-dependent manner, which occurred at the same time as the fluorescence intensity of p-YB-1 increased (Fig. 4K–L). The results of the EdU assay showed that LJI308 treatment induced DNA replication reduction, which may be attributed to cell cycle arrest. The lower fluorescence intensity represented attenuated DNA replication, and DNA replication stagnation was induced by the combined treatment of abemaciclib and LJI308 (Fig. 4M–N).

### YB-1 phosphorylation at S102 confers CDK4/6i resistance

**Fig. 5:**
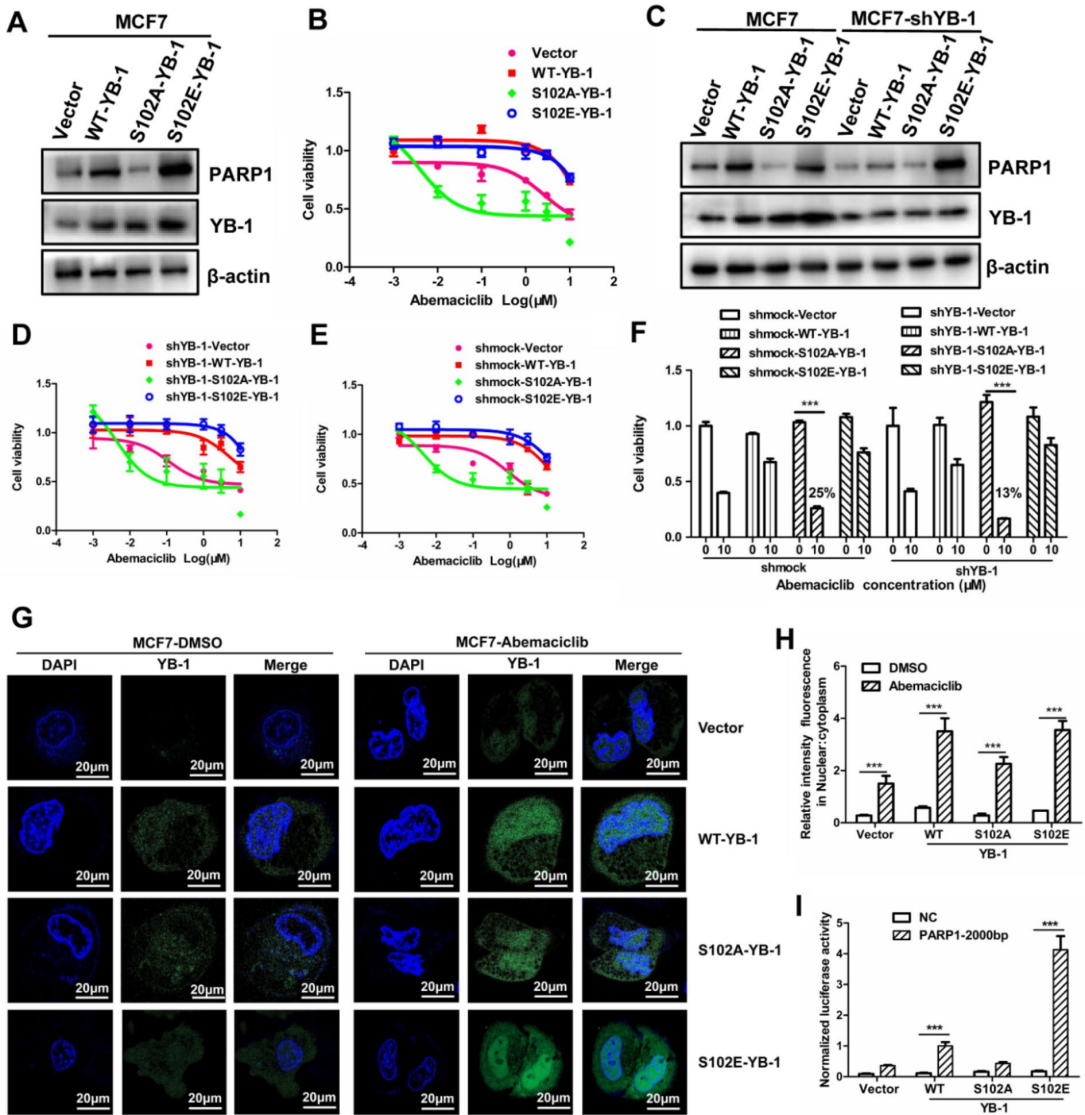
YB-1 phosphorylation at S102 confers CDK4/6i resistance. **A**. The plasmids YB-1-S102A and YB-1-S102E were used to simulate dephosphorylation and hyperphosphorylation, respectively, and two plasmids were transfected separately into MCF7 cells. The PARP1 level was almost invisible in the YB-1-S102A group and was significantly increased in the YB-1-S102E group compared to the wild-type YB-1 group, as the Western blot data show. **B**. The fluctuation of CDK4/6i sensitivity was confirmed by MTT assay when cells were transfected with YB-1-S102A and YB-1-S102E plasmids. **C**. The two mutant YB-1 plasmids were transfected into MCF7-shYB-1 cells; the Western blot data showed that the PARP1 level in the shYB-1 group was lower than that in the control group. **D–E**. The change in CDK4/6i sensitivity for mutant YB-1 plasmid transfection was confirmed by MTT assay. **F**. The inhibition efficiency of cell viability for the YB-1-S102A-MCF7-shYB-1 group reached approximately 13% when transfected with the mutant YB-1 plasmid when selecting the cell viability of abemaciclib at 10 μM, **G–H**. The YB-1 nuclear translocation was increased under abemaciclib treatment, as determined by an immunofluorescence assay. **I.** We constructed an upstream 2000 bp fragment in the promoter of PARP1 into a luciferin vector plasmid for a double fluorescein reporter gene assay. The effect of mutant plasmid transfection on PARP1 transcription was determined by the change in fluorescence intensity under transfection with the YB-1-WT/S102A/S102E plasmids. The results presented have been repeated in 3 biological replicates. Data, means ± SEMs, *, P < 0.05, **, P < 0.01, ***, P < 0.001

We found that YB-1 was phosphorylated at S102, was critical for PARP1 transcriptional activation and was associated with CDK4/6i resistance. The mutant plasmids YB-1-S102A and YB-1-S102E were constructed to study the importance of YB-1 phosphorylation. The plasmids YB-1-S102A and YB-1-S102E were used to simulate dephosphorylation and hyperphosphorylation, respectively. The two plasmids were transfected separately into MCF7 cells in which YB-1 was expressed at low levels. There was no increase in the protein level of PARP1 in the YB-1-S102A group compared to the wild-type YB-1 group; in contrast, the PARP1 level was significantly increased in the YB-1-S102E group (Fig. 5A). The MTT assay indicated that YB-1-S102A transfection did not lead to CDK4/6i resistance. Furthermore, YB-1-S102E transfection greatly enhanced CDK4/6i resistance (Fig. 5B). Considering the low level of YB-1 expression in MCF7 cells, we transfected two mutant YB-1 plasmids into MCF7-shYB-1 cells. We discovered that YB-1 activity correlated positively with PARP1, and the PARP1 level in the shYB-1 group was lower than that in the control group (Fig. 5C). Moreover, the inhibition efficiency of cell viability for the YB-1-S102A-MCF7-shYB-1 group reached approximately 13% when transfected with the mutant YB-1 plasmid (Fig. 5D–F).

We found that PARP1 and p-YB-1 were colocalized in the nucleus (Fig. 4G). The protein level of p-YB-1 gradually increased under abemaciclib treatment, which was determined by immunofluorescence assay. The phenomenon of YB-1 nuclear translocalization was barely observed in the YB-1-S102A transfection group when treated with abemaciclib. In contrast, YB-1 nuclear translocation was clearly observed in the YB-1-WT and YB-1-S102E groups, which conferred CDK4/6i resistance. These results reminded us that YB-1 nuclear translocation may be closely correlated with CDK4/6i resistance and may be the driving and critical factor for the development of drug resistance (Fig. 5G–H). To further determine the effect of mutant plasmid transfection on PARP1 transcription, we constructed an upstream 2000 bp fragment in the promoter of PARP1 into a luciferin vector plasmid for a double fluorescein reporter gene assay. The change in fluorescence intensity can be used as the basis of judgment of the effect on PARP1 transcription under transfection with the YB-1-WT/S102A/S102E plasmids. The fluorescence intensity in the YB-1-WT and YB-1-S102E groups was higher than that in the YB-1-S102A group, which barely worked. PARP1 transcription may be influenced by YB-1 phosphorylation at S102. (Fig. 5I). Taking the previously mentioned results together, we can conclude that YB-1 phosphorylation can promote its nuclear translocation and in turn lead to transcriptional activation of PARP1, eventually leading to the occurrence of CDK4/6i resistance.

### Interrupting the "YB-1-PARP1" loop enhanced the tumor-killing effects of CDK4/6i.

**Fig. 6:**
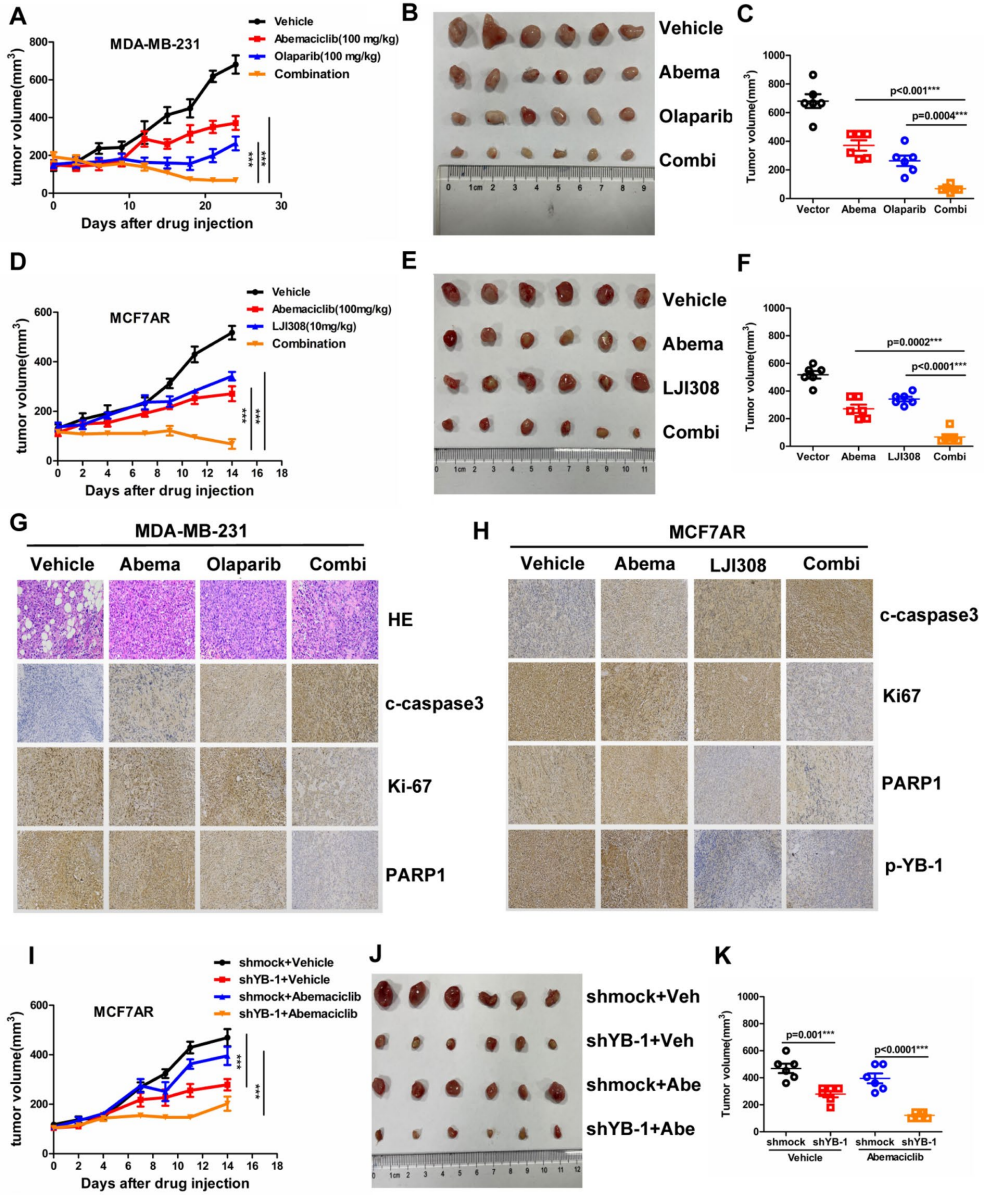
Interrupting the "YB-1-PARP1" loop enhanced the tumor-killing effects of CDK4/6i. **A**. MDA-MB-231 cells were used to explore the effects of combined therapy in the nude mouse model. The mice were randomly divided into vehicle, abemaciclib, olaparib and combined groups when the tumor size reached 150 mm^3^. At least 6 nude mice were included in each group, and the nude mice were injected by intragastric administration 3 times a week with 100 mg/kg. The combined therapy of abemaciclib and olaparib synergistically suppressed tumor growth in the xenograft model. **B–C**. Tumor shape and size are shown for the 4 groups, and a ruler was used to determine the tumor volume. A comparison of tumor volume between groups is shown. **D**. The YB-1 inhibitor LJI308 combined with abemaciclib exerted a synergistic antitumor effect in MCF7AR cells, causing the xenograft tumor to shrink rapidly. **E–F**. Tumor shape and size are shown for the 4 groups, and a ruler was used to determine the tumor volume. A comparison of tumor volume between groups is shown. **G**. The sample derived from the animal tumor tissue was used to detect c-c3 (cleaved-caspase3), Ki67, and PARP1 expression in 4 groups. **H**. The IHC results of c-c3, Ki67, PARP1 and p-YB-1 are displayed for the effects on PARP1 expression induced by YB-1 inhibition. **I–J** YB-1 knockdown slowed the rate of tumor growth under abemaciclib treatment, and changes in tumor volume and tumor size are shown. The results presented have been repeated in 3 biological replicates. Data, means ± SEMs, *, P < 0.05, **, P < 0.01, ***, P < 0.001

The ‘‘YB-1-PARP1’’ loop was proven to play a crucial role in the CDK4/6i resistance of breast cancer, and blocking the factor in the ‘‘YB-1-PARP1’’ loop can enhance the sensitivity of CDK4/6i efficiency. MDA-MB-231 and MCF7AR cells were used for the nude mouse model. The mice were randomly divided into vehicle, abemaciclib, olaparib and combined groups when the tumor size reached 150 mm^3^. At least 6 nude mice were included in each group, and the nude mice were intragastrically injected with 100 mg/kg abemaciclib or olaparib and intraperitoneally injected with 10 mg/kg LJI308 three times a week. The combined therapy of abemaciclib and olaparib synergistically suppressed tumor growth in the xenograft model (Fig. 6A–C). In addition, the YB-1 inhibitor LJI308 combined with abemaciclib exerted a synergistic antitumor effect, causing the xenograft tumor to shrink rapidly. Abemaciclib or LJI308 was intraperitoneally injected i.p. 3 times a week with a 100 mg/kg dose (Fig. 6D–F). The sample derived from the animal tumor tissue in the previously mentioned Experiment A was used to detect c-caspase3, Ki67, and PARP1 expression in the 4 groups (Fig. 6G). The IHC results of c-c3, Ki67, PARP1 and p-YB-1 in Experiment B are displayed for the effects on PARP1 expression induced by YB-1 inhibition (Fig. 6H). Simultaneously, we discovered that YB-1 knockdown slowed the rate of tumor growth under abemaciclib treatment (Fig. 6I–K).

### Low "p-YB-1-PARP1" expression was linked to CDK4/6i sensitivity in breast cancer

**Fig. 7:**
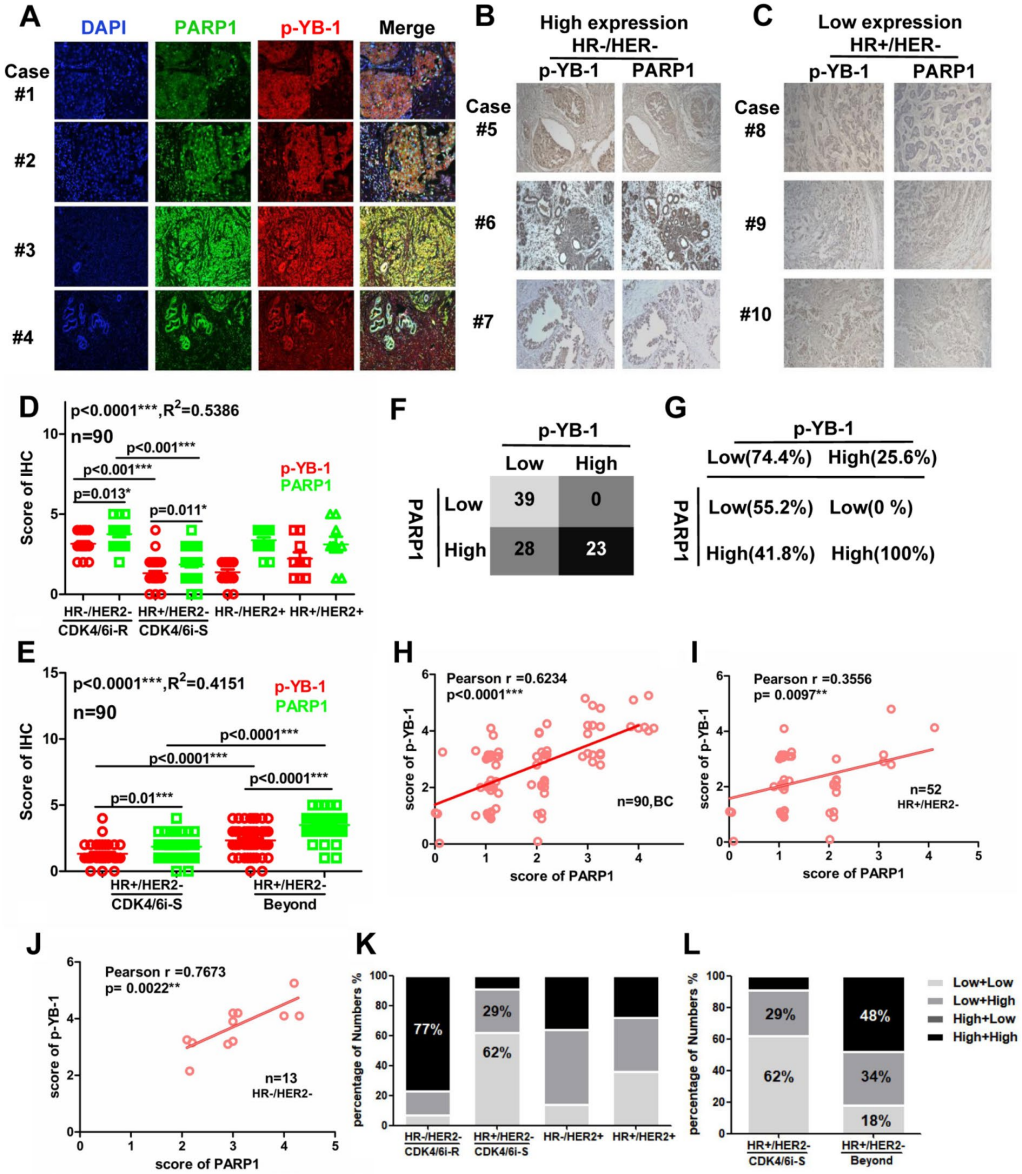
Low ‘‘p-YB-1-PARP1’’ expression was linked to CDK4/6i sensitivity in breast cancer. **A**. The data from multiple immunofluorescence (MIF) assays indicated that p-YB-1 and PARP1 were coexpressed and colocalized in four representative breast cancer patients. The paraffin sections of the breast cancer patients were separately stained with p-YB-1 (red), PARP1 (green) and DAPI (blue). **B–C** The samples from 80 breast cancer patients were used for detection of p-YB-1 and PARP1 by IHC assay, the staining score ranged from 0 to 5. The 80 breast cancer patients were divided into HR + /HER2-, HR-/HER2-, HR-/HER2 + and HR + /HER2 + groups. **D–E** Typical images of high and low ‘‘p-YB-1/PARP1’’ expression in HR-/HER2- and HR + /HER2- patients are shown, and the staining results came from the same position of successive slices. **F–G** The patients were divided into four groups: p-YB-1^L^ + PARP1^L^, p-YB-1^L^ + PARP1^H^, p-YB-1^H^ + PARP1^L^, and p-YB-1^H^ + PARP1^H^ groups, based on the expression of p-YB-1 and PARP1. The percentage of the high PARP1 group accounted for 100% of the high p-YB-1 group. **H–J** The scatter plots of the data clearly indicated that PARP1 positively correlated with p-YB-1 in the BC (n = 90, r = 0.6234, p < 0.0001***), HR + /HER2- (n = 52, r = 0.3556, p = 0.0097**) and HR-/HER2-(n = 13, r = 0.7673, p = 0.0022**) groups. **K–L** Patients in the low p-YB-1 group accounted for approximately 91% of CDK4/6i-sensitive clinical patients. In contrast, the patients with high p-YB-1 levels accounted for approximately 77% of CDK4/6i-resistant TNBC clinical patients. The results presented have been repeated in 3 biological replicates. Data, means ± SEMs, *, P < 0.05, **, P < 0.01, ***, P < 0.001.

Although abemaciclib has shown efficacy in HR + /HER2- breast cancer patients, many patients demonstrate intrinsic or acquired resistance to CDK4/6i and disease progression [42]. We collected paraffin sections from breast cancer patients from the First Affiliated Hospital of Shengzhen University. Previous research has revealed the relationship of p-YB-1 and PARP1 in cell line experiments, but the expression and colocalization of p-YB-1/PARP1 in breast cancer tissues are unknown. The data from multiple immunofluorescence (MIF) assays indicated that p-YB-1 and PARP1 were coexpressed and colocalized in four representative breast cancer patients. The codistribution of p-YB-1 and PARP1 has obvious characteristics of regional consistency (Fig. 7A). Generally, the patients expressing HR + /HER2- tended to be sensitive to CDK4/6i, so that most of this group can benefit potentially from CDK4/6i therapy. The IHC results showed lower ‘‘p-YB-1/PARP1’’ expression in patients with HR + /HER2- status than in patients with other HR-/HER2-, HR-/HER2 + and HR + /HER2 + statuses. The HR-/HER2- group, as the triple-negative breast cancer type, was considered resistant to CDK4/6i with higher ‘‘p-YB-1/PARP1’’ expression. To increase the accuracy of the results, the staining results came from the same position of successive slices, and typical images are shown (Fig. 7B–C). By further analyzing the correlation of p-YB-1 and PARP1 in HR + /HER2- and non-HR + /HER2- group patients, the data suggested that p-YB-1 was positively correlated with PARP1 in both the HR + /HER2- and non-HR + /HER2- groups and discovered that there were great differences in p-YB-1/PARP1 expression between the HR + /HER2- and non-HR + /HER2- groups (Fig. 7D–E). ‘‘p-YB-1/PARP1’’ expression remained consistently high in HR-/HER2-and low in HR + /HER2- patients. According to the expression of p-YB-1 and PARP1, we divided samples into four groups: p-YB-1^L^ + PARP1^L^, p-YB-1^L^ + PARP1^H^, p-YB-1^H^ + PARP1^L^, and p-YB-1^H^ + PARP1^H^. Surprisingly, the percentage of the high PARP1 group accounted for 100% of the high p-YB-1 group, which indicated that PARP1 was almost highly expressed when p-YB-1 was highly expressed (Fig. 7F–G). Furthermore, the scatter plots of the data clearly indicated that PARP1 positively correlated with p-YB-1 in the BC (n = 90, r = 0.6234, p < 0.0001***), HR + /HER2- (n = 52, r = 0.3556, p = 0.0097**) and HR-/HER2- (n = 13, r = 0.7673, p = 0.0022**) groups of patients (Fig. 7H–J). CDK4/6i-sensitive clinical patients mainly included the p-YB-1^L^ + PARP1^L^ and p-YB-1^L^ + PARP1^H^ groups, which accounted for approximately 91%. In contrast, the patients with HR-/HER2- status were all ascribed to the p-YB-1^H^ + PARP1^L^ and p-YB-1^H^ + PARP1^H^ groups, which accounted for approximately 77% (Fig. 7K–L). Based on these results, we concluded that low ‘‘p-YB-1/PARP1’’ was linked to CDK4/6i sensitivity. The PARP1 level positively correlated with p-YB-1. Thus, taking the level of ‘‘p-YB-1/PARP1’’ into consideration is feasible when assessing the benefit of CDK4/6i therapy for patients.

### Working model of the “YB-1-PARP1” pathway involved in CDK4/6i resistance

**Fig. 8:**
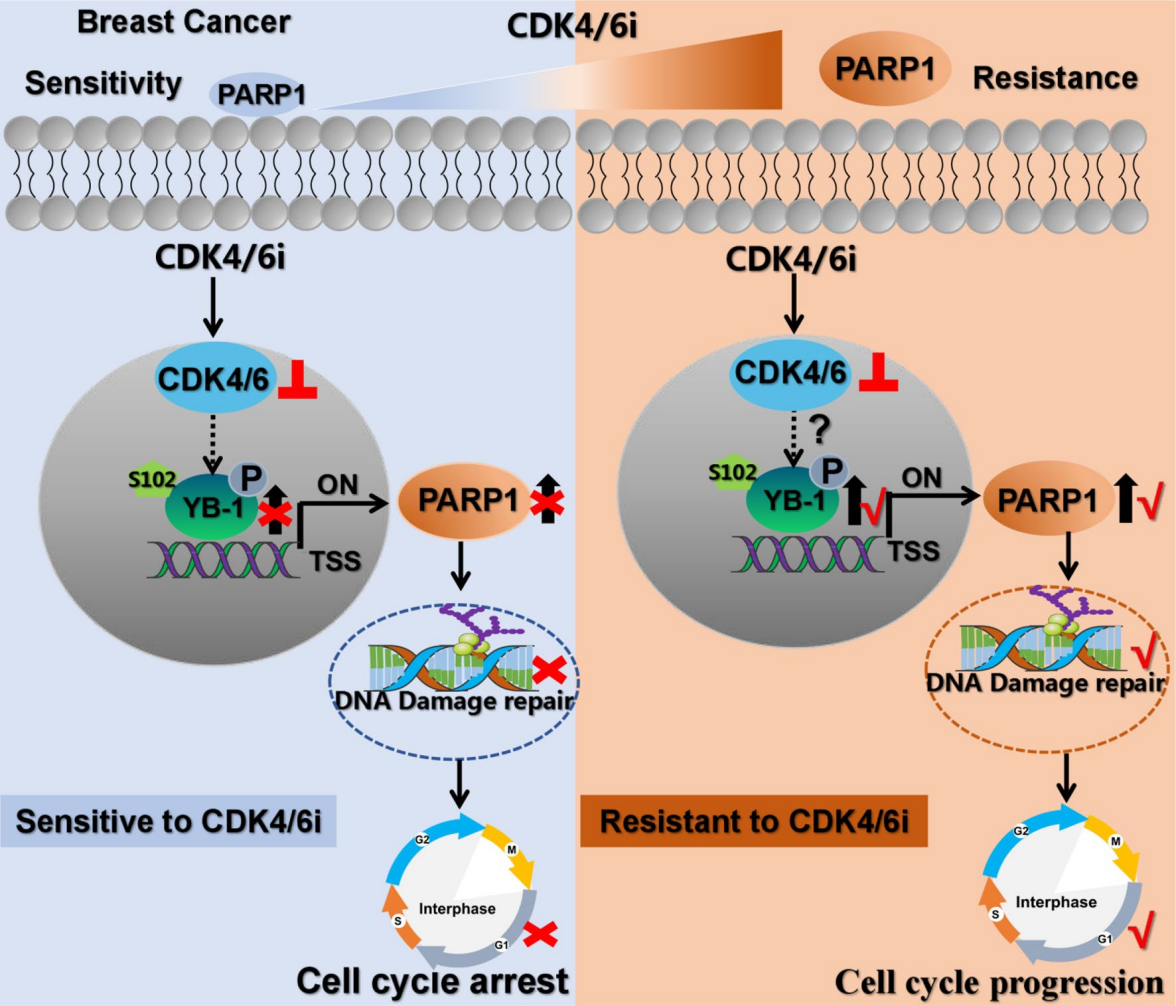
Working model of the “YB-1-PARP1” pathway involved in CDK4/6i resistance. CDK4/6i-resistant breast cancer cells display elevated PARP1 expression and YB-1 activation. CDK4/6i treatment may promote YB-1 phosphorylation at S102, and enhanced YB-1 phosphorylation leads to the binding of YB-1 to the promoter region of PARP1, which results in transcriptional activation of PARP1. Phosphorylated YB-1 can control cell cycle progression by strengthening PARP1-mediated DNA damage repair. Increasing PARP1 is crucial for promoting the cell cycle progression, which is the key role of PARP1 in removing the blocking effect of CDK4/6i on G1/S transformation of cells. The role of bypass activation of the cell cycle in PARP1 upregulation and YB-1 phosphorylation is crucial for CDK4/6i resistance

To better understand the molecular mechanism of this paper, we have drawn a schematic model summarizing how PARP1 orchestrates CDK4/6i resistance by promoting DNA damage repair and driving the cell cycle G1/S phase transition. Elevated p-YB-1 enhances the binding of YB-1 to the promoter region of PARP1 when CDK4/6i treatment occurs, and PARP1 is transcriptionally activated by YB-1 phosphorylation. High levels of PARP1 are closely associated with CDK4/6i resistance, and phosphorylated YB-1 can control cell cycle progression by strengthening PARP1-mediated DNA damage repair. YB-1 phosphorylation may remove the blocking effect of CDK4/6i on the G1/S phase transition and lead to the occurrence of CDK4/6i resistance in breast cancer. The role of bypass activation of the cell cycle in PARP1 upregulation and YB-1 phosphorylation was crucial for CDK4/6i resistance. Fig. 8.

## Discussion

Despite these favorable outcomes, not all patients benefit from CDK4/6i inhibition, and most patients who do initially respond will ultimately progress. It is essential to study acquired resistance to CDK4/6i to gain perspective into the mechanism behind persistent resistance in BC. Deciphering the molecular features that determine sensitivity and resistance to CDK4/6i is crucial to identifying predictive biomarkers and novel therapeutic targets for improving CDK4/6i efficacy and patient outcomes.

A great number of targets and inhibitors have been discovered, with the aim of resolving the CDK4/6i-resistance problem [7–9, 13, 14]. In this study, we sought to investigate the molecular profiles associated with intrinsic and acquired resistance to CDK4/6i in HR + /HER2- BC. We focused on the transcription factors YB-1 and KLF5 [19, 20] to determine whether these proteins can play critical roles in CDK4/6i resistance. To further understand how molecules evolve during the development of CDK4/6i resistance, we carried out experiments on CDK4/6i-sensitive MCF7 cells to construct resistant strains, which were established by treatment with a 6-month dose of abemaciclib. Surprisingly, significantly higher ‘‘p-YB-1-PARP1’’ expression was observed in MCF7AR cells than in parental cells. We also found that CDK4/6i treatment led to PARP1 upregulation in breast cancer patients and cells. Furthermore, the results from cell assays and the xenograft model indicated that PARP1 or YB-1 inhibition restores sensitivity to CDK4/6i. We conclude that YB-1 can regulate the level of PARP1 protein through transcriptional regulation, which relies on YB-1 phosphorylation and translocation from the cytoplasm to the nucleus. The synergistic antitumor effect of the abemaciclib and olaparib combination in vitro and in vivo suggested that PARP1 inhibition can reverse CDK4/6i resistance, which may bring about new therapeutic strategies. Olaparib was approved by the American FDA for the treatment of high-risk early-stage breast cancer patients with HER2-positive and BRCA mutations in 2022, which guaranteed the rationality of the combination therapy. Olaparib may be recommended for consideration when PARP1 is upregulated in CDK4/6i-resistant patients. To the end, the results from the patient samples showed that low ‘‘p-YB-1-PARP1’’ is linked to CDK4/6i sensitivity, and the levels of ‘‘p-YB-1 and PARP1’’ may be taken into consideration when assessing the benefit of CDK4/6i therapy for patients.

Taken together, our results suggest a role for activation of the ‘‘p-YB-1/PARP1’’ pathway in promoting acquired resistance to CDK4/6i in BC. Integrating these data, some problems should be further studied and verified. First, we sought to determine whether cell cycle progression is closely related to YB-1 phosphorylation. It has been reported that YB-1 expression is associated with poor prognosis in primary melanoma patients, which is primarily dependent on S102 unphosphorylated cytoplasmic YB-1. The cytoplasmic activity of YB-1 stimulates the tumorigenicity and metastatic potential of melanoma cells by promoting EMT-like properties [43]. Another paper described that S102 phosphorylated YB-1 enhanced its cellular localization [36]. Doxorubicin treatment, which caused the resultant G2 arrest during the cell cycle, led to a significant increase in the number of cells where YB-1 was not found in the cytoplasm. S102 unphosphorylated YB-1 moves to the cytoplasmic compartment when the cell cycle is completed. We can conclude that the cell cycle is closely related to the YB-1 phosphorylation state. S102 phosphorylated YB-1 is crucial for promoting the cell cycle moving forward, which is the key role PARP1 plays in removing the blocking effect of CDK4/6i on G1/S transformation of cells. Phosphorylated YB-1 can control cell cycle progression by strengthening PARP1-mediated DNA damage repair. Taken together, these results indicate that the role of bypass activation of the cell cycle in YB-1 phosphorylation is crucial for CDK4/6i resistance. In another paper, it was reported that YB-1 phosphorylation induced by ionizing radiation enhances DNA-DSB repair, which may reveal that YB-1 phosphorylation promotes DNA damage repair by PARP1 [35]_**.**_ The role of YB-1 phosphorylation in mediating drug resistance has been reported in sorafenib-resistant hepatocellular carcinoma cells and diffuse large B-cell lymphoma [30, 32, 33]. The literature indicates that RSK can mediate cell cycle progression, which is dependent on YB-1 phosphorylation [44]. LJI308, an inhibitor of YB-1 phosphorylation, was proven to function as a blocker of DNA damage repair [34].

Second, whether the nuclear translocation from the cytoplasm mediated by YB-1 phosphorylation is a key driver of CDK4/6i resistance remains unclear. It has been reported that the activity of the cytoplasmic retention site (CRS, a.a. 267–293) prevails over the nuclear localization signal (NLS, a.a. 186–205), which results in the predominantly cytoplasmic localization of YB-1 [26, 45]. YB-1 translocates to the nucleus at the boundary of the G1/S phase [26]. Akt phosphorylates the Y-box at Ser102 and promotes its nuclear translocation, which affects the expression of drug-resistance genes and other genes associated with malignant characteristics in ovarian cancer cells [46, 47]. Next, the truncated YB-1 plasmid without an NLS signal will be transfected into cells, and whether YB-1 nuclear translocation is a key driver of CDK4/6i resistance will be tested. The literature indicates that YB-1 nuclear translocation is negatively regulated by S209 phosphorylation even in the presence of phosphorylated S102 [48]. Taken together, these data indicate that the nuclear localization of YB-1 may be the driver of CDK4/6i resistance.

Last, it was found that some triple-negative breast cancer (TNBC) cell lines showed sensitivity to palbociclib with obvious growth inhibition, and the efficacy of palbociclib on TNBC was irrespective of the status of HR and HER2 [49]. Selective CDK4/6 inhibitors have demonstrated potential therapeutic effects on TNBC [50, 51]. There is evidence to show that the efficacy of CDK4/6i largely depends on the expression and activation status of proteins such as Rb, PI3K, and EGFR [52, 53]. It has been confirmed that TNBC cell lines with high Rb gene expression are sensitive to CDK4/6i and are blocked in the G1 phase, while TNBC cell lines with Rb gene mutations or low Rb gene expression are not arrested in the cell cycle [50, 51]. Twelve TNBC cell lines with wild-type Rb showed high sensitivity to CDK4/6 inhibitors [52]. Some TNBC cells with high CDK2 expression were resistant to CDK4/6i treatment, and entry into the cell cycle was dependent on Rb phosphorylation by CDK2, which can bypass the CDK4/6-independent G1/S transition induced by CDK4/6i treatment. Therefore, more genetic backgrounds, such as Rb, PARP1 and p-YB-1, may need to be checked when evaluating abemaciclib effects, which are not restricted by HR/HER2 status. Our results from the SK-BR-3(HER2 +) and MDA-MB-231(TNBC) cell lines all showed that YB-1 inhibition can enhance the efficacy of abemaciclib treatment. It has been reported that YB-1 phosphorylation is consistent with G1-S transition synchronization during cell cycle phases [36]. Additionally, p-YB is overexpressed in TNBC cell lines and clinical samples. Comprehensively taking into account the function of YB-1 phosphorylation in promoting DSB repair and driving the cell cycle G1/S phase transition, we hypothesized that YB-1 phosphorylation may remove the blocking effect of CDK4/6i on the G1/S phase transition and lead to the occurrence of CDK4/6i resistance in breast cancer. Therefore, the level of p-YB-1 could be used as a predictive marker for CDK4/6i efficacy in TNBC.

Other pathways may be regulated by YB-1 phosphorylation, which leads to CDK4/6i resistance. It has been reported that YB-1 phosphorylation promotes YB-1 degradation through ubiquitination [54], and this phenomenon is not consistent with our result of the downregulation of YB-1 when inhibited by LJI308. This mechanism may be the negative regulatory signal that controls cell cycle signals. Ubiquitination may be attenuated in CDK4/6i-resistant cells, and strengthening ubiquitination may be a solution for CDK4/6i tolerance. The literature clearly shows that S102 phosphorylation of YB-1 may mediate trastuzumab resistance by increasing CD44 stem cells. Targeting YB-1 can eliminate the unresponsive TIC population and render the cancer sensitive to therapy [55]. Another paper noted that S102 phosphorylation of YB-1 increased CD44 expression growth and enhanced tumor growth by mammosphere formation [56]. The data from the cBioportal database show that HR + /HER2- patients with PARP1 amplification have lower overall survival times. Meanwhile, patients with higher YBX1 levels had a lower survival rate. Surprisingly, there was no significant difference between the patients with high or low PARP1, and the function of PARP1 in breast cancer may be related to the mutation status of the BRCA gene. These works indicate that YB-1 phosphorylation may be involved in tumor cell stemness, which is closely related to tumor drug resistance. Related research work is underway to resolve the problems mentioned above. However, this study is innovative in that we evaluated the synergetic effect of CDK4/6i combined with new YB-1 inhibitors in preclinical models.

### Supplementary Information

**Additional file 1: Table S1.** The pathological information of clinical breast cancer patients.

**Additional file 2: Table S2.** The information of antibody used for WB, IHC and IF.

**Additional file 3: Table S3.** The primers lists.

## Data Availability

The mentioned datebase website in this paper were listed as http://gepia.cancer-pku.cn/, https://www.cancerrxgene.org/, and https://sites.broadinstitute.org/ccle.
